# Asthma severity in four countries of Latin America

**DOI:** 10.1186/s12890-019-0871-1

**Published:** 2019-07-09

**Authors:** Hugo Neffen, Felipe Moraes, Karynna Viana, Valentina Di Boscio, Gur Levy, Claudia Vieira, Gabriela Abreu, Claudia Soares

**Affiliations:** 1 Centro de Alergia e Inmunología - Santa Fe, Santa Fe, Argentina; 2 GSK, Rio de Janeiro, Brazil; 3 GSK, Buenos Aires, Argentina; 4 GSK, Ciudad de Panamá, Panamá

**Keywords:** Asthma, Severity, Epidemiology, Disease management, Latin America

## Abstract

**Background:**

In Latin America, there is scarce information about severe asthma (SA) according to the ERS/ATS 2014 criteria. This study aimed to compare the demographic, socio, clinical characteristics, treatment, and use of healthcare resources between SA and non-severe asthma (NSA) patients in Argentina, Colombia, Chile and Mexico.

**Methods:**

A cross-sectional study was conducted including 594 asthma patients from outpatient specialized sites. A descriptive analysis was performed comparing SA patients and NSA. Chi-square and Mann Whitney tests were used to assess associations between asthma severity and outcome variables.

**Results:**

Using ERS/ATS 2014 criteria, 31.0% of the patients were identified as SA. SA patients were older at diagnosis (mean age 31.64 years vs 24.71 years, *p* < 0.001) and had higher proportion of uncontrolled asthma than the NSA patients (64.1% vs 53.2%, *p* < 0.001). SA patients reported a significantly higher proportion of both hospital admission and emergency room (ER) visits due to asthma in the last year, compared with NSA patients, 8.7% vs. 3.7% (*p* = 0.011) and 37.0% vs. 21.7% (*p* < 0.001), respectively.

**Conclusions:**

SA patients were older, had greater proportions in some comorbidities and experienced increased healthcare utilization. Also, our results showed that even in patients using the last steps of treatment (GINA step 4 or 5), there was still a higher proportion of uncontrolled disease.

**Electronic supplementary material:**

The online version of this article (10.1186/s12890-019-0871-1) contains supplementary material, which is available to authorized users.

## Background

Asthma is a chronic inflammatory disorder of the airways [1] and its prevalence is estimated to range from 0.2 to 21.0% globally [2]. It is one of the most common chronic diseases among children [3] and young adults [2], and it is a significant cause of disability, high health resource utilization, and poor quality of life for those who are affected [1]. Thus, it accounts for considerable healthcare costs and loss of work productivity [4, 5].

Approximately 2–10% have some form of severe asthma (SA) [6, 7]. According to ERS/ATS 2014 guidelines [8], SA is defined as asthma that requires step four or five treatment (e.g. high-dose Inhaled Corticosteroids (ICS)/Long-acting beta2-agonist (LABA)) to prevent it from becoming ‘uncontrolled’, or asthma that remains ‘uncontrolled’ despite this treatment. SA is a heterogeneous disease with high variability in clinical presentation, physiological characteristics, and disease manifestation [6, 9].

In Latin America, there is a lack of information related to SA epidemiology and the burden of SA following implementation of the ERS/ATS 2014 criteria. Essentially, most of the Latin American studies that have assessed the SA population in the region had also included untreated patients [10, 11] or focused on the, uncontrolled asthma population [12]. Therefore, by using the ERS/ATS 2014 criteria the aims of this study were to compare the demographic, socioeconomic, and clinical characteristics, as well as treatment courses and use of healthcare resources between SA patients and non-severe asthma (NSA) patients in Argentina, Colombia, Chile, and Mexico.

## Methods

### Study design and population

The Asthma Control in Latin America (ASLA) study was a cross-sectional multisite study. Patients were consecutively enrolled between December 2013 and December 2015. The 16 recruiting sites were located in Argentina (five sites); Chile (five sites), Colombia (three sites), and Mexico (three sites) [13, 14].

The study included 594 diagnosed and treated asthma patients who were recruited during routine care at specialized outpatient sites, aged over 12 years old, under pneumologists follow up, with at least one prescription of asthma medication and one medical visit for asthma within the last 6 months. Individuals were excluded if they were participating in a clinical trial at the time of the study or were unable or not willing to comply with the study requirements. This study was conducted in accordance with the amended Declaration of Helsinki [15]. Written informed consent was obtained from all the participants. Minors younger than 18 years old signed the assent form and parents or legal guardians provided their written informed consent. Institutional review boards in each country approved consent forms and procedures.

### Measurements

During the course of a scheduled medical visit, the participating patients were asked to complete the Asthma Control Test (ACT) [16], which was a self-administered questionnaire containing five items that can each be rated on a five-point Likert scale. Based on the ACT score, subjects were categorized as controlled (ACT score ≥ 20) or uncontrolled (ACT score < 20). The physician conducted an interview, which included questions on socio-demographic data [gender, age, skin colour (white, African-Latin American, Native American, multiple and other skin colours that were not listed)]; healthcare utilization in the last year; treatment used; and comorbidities.

Asthma severity was operationally defined using an algorithm based on answers to questions relating to the ERS/ATS 2014 criteria. SA was defined as asthma that required step four or five treatment (i.e.: high ICS doses plus a second controller or use of OCS (oral corticosteroid) regardless of ICS doses). Four treatment categories did not fulfil the ERS/ATS 2014 criteria for SA and NSA and were reviewed by a panel of three pneumologist experts on asthma to allocate a group (Table 1). Clinically significant asthma exacerbations were based on patients self-reported healthcare resource utilization, and were defined as any emergency room (ER) visit or hospitalization due to asthma.

**Table 1:** Respirologist-adjudicated decisions about patients’ asthma classifications and treatments, for cases where ERS/ATS 2014 guidelines were unclear

Treatment scheme not clearly defined by ERS/ATS 2014	Classification decision – defined by 3 respirologists
1: Patients using non-high dose of ICS + LAMA	Severe asthma
2: Patients using non-high dose of ICS + LABA+Anti-IgE	Severe asthma
3: Patients using OCS with other controllers that are not ICS	Severe asthma
4: Patients using non-high dose of ICS + 2 or more controllers *(leukotrienes receptor antagonist OR LABA OR xanthines (theophylline OR aminophylline)*	Non-severe asthma

*ICS* inhaled corticosteroids, *LAMA* long-acting muscarinic receptor antagonist, *LABA* long-acting beta2 -agonist, *Anti-IgE* anti-immunoglobulin E, *OCS* oral corticosteroid

### Data Analysis

A descriptive analysis was carried out, comparing socio-demographic characteristics, clinical factors, asthma control, nutritional status, healthcare utilization in the last year, treatment used, and comorbidities of SA patients compared with NSA patients.

Body Mass Index (BMI) was calculated in adults as weight divided by the square of height, and the nutritional status categorized as: underweight < 18.5 Kg/m^2^; eutrophic = 18.5–24.9 Kg/m^2^; overweight = 25–29.9 Kg/m^2^; and obesity ≥30Kg/m^2^. BMI in adolescents was calculated following the indications described elsewhere [17] and the nutritional status classified as underweight (Z-score < − 3 and < − 2); eutrophic (Z-score ≥ − 2 and ≤ 1); overweight (score-Z > 1 and ≤ 2); and obese (Z-score > 2). For demographic and clinical outcome variables, Chi-square test or the Fisher exact test (as appropriate) and Mann Whitney test were used to assess associations between SA patients and NSA patients. Values of *p* < 0.05 were considered statistically significant. Data analysis was performed using Stata™13 College Station,TX: StataCorpLP and SPSS™ version 24.

## Results

A total of 594 asthma patients were studied: 154 subjects from Argentina, 154 from Chile, 163 from Mexico and 123 from Colombia. Overall 72.7% were women and 50.8% white, with a median age of 47 years old at the time of the study visit and an age of 25 at the time of asthma diagnosis. According to the ERS/ATS 2014 criteria, 31.0% of the patients had SA (Table 2). Among the SA patients, 88.0% were classified as SA by the use of high ICS dose plus a second controller (Table 4). Of the 12% remaining, about 11.4% used ICS in doses that were not high but were classified as SA due to the use of anti-IgE, tiotropium, anti-IgE and tiotropium at the same time or OCS. The last 0.6% used OCS without ICS in combination and due to that it was also considered as SA.

Differences in socio-demographic and clinical factors by SA and NSA are shown in Table 2. NSA patients were younger than the SA patients at study visit and at diagnosis. 72.1% of the SA patients were overweight or obese. A higher proportion of SA patients reported having performed at least one PEF test in the last year compared with NSA patients (35.9% vs. 16.6%, *p* < 0.001).

**Table 2:** Sociodemographic and clinical factors by severe asthma status in Argentina, Chile, Colombia, and Mexico 2013–2015

Independent variables	Severe asthma		Non-severe asthma		*p*-value	Total	
	N	(%)	N	(%)		N	(%)
Gender							
Male	44	23.9	118	28.8	0.218	162	27.3
Female	140	76.1	292	71.2		432	72.7
Age at the moment of medical visit (years)							
12–19	11	6.0	50	12.2	< 0.001	61	10.3
20–29	14	7.6	64	15.6		78	13.1
30–39	13	7.1	70	17.1		83	14.0
40–49	29	15.8	83	20.2		112	18.9
50–59	53	28.8	67	16.3		120	20.2
≥ 60	64	34.8	76	18.5		140	23.6
Age (years)							
Mean (±SD)	51.81 (±16.76)		42.36 (±18.03)		< 0.001	45.29 (±18.16)	
Median (IQR)	54 (20)		42 (28)			47 (28)	
Age at asthma diagnosis (years)							
≤ 9	39	21.2	112	27.3	< 0.001	151	25.4
10–25	37	20.1	116	28.3		153	25.8
26–41	42	22.8	104	25.4		146	24.6
≥ 42	66	35.9	78	19.0		144	24.2
Age at asthma diagnosis (years)							
Mean (±SD)	31.64 (±20.94)		24.71 (±18.27)		< 0.001	26.86 (±19.38)	
Median (IQR)	30 (38)		23 (30)			25 (32)	
Reason for the medical visit							
Obtain prescription	2	1.1	8	2.0	0.820	10	1.7
Routine check-up	181	98.4	398	97.1		579	97.5
Other	1	0.5	4	1.0		5	0.8
Race/Ethnicity^a^							
White	103	56.3	198	48.3	< 0.001	301	50.8
African-Latin American	34	18.6	184	44.9		218	36.8
Native American	3	1.6	5	1.2		8	1.4
Other	4	2.2	2	0.5		6	1.0
Multiple	39	21.3	21	5.1		60	10.1
Nutritional status^b^							
Underweight	1	0.5	13	3.2	0.005	14	2.4
Eutrophic	50	27.3	152	37.1		202	34.1
Overweight	73	39.9	154	37.7		227	38.3
Obesity	59	32.2	90	22.0		149	25.2
Had at least one PEF test last 12 months							
Yes	66	35.9	68	16.6	< 0.001	134	22.6
No	118	64.1	342	83.4		460	77.4
Had at least one spirometry last 12 months							
Yes	133	72.3	264	64.4	0.059	397	66.8
No	51	27.7	146	35.6		197	33.2

*BMI* body mass index, *PEF* Peak Expiratory Flow, *USD* US dollars, *SD* Standard deviation, *IQR* interquatile range

^a^1 missing value

^b^2 missings values

**Fig. 1:**
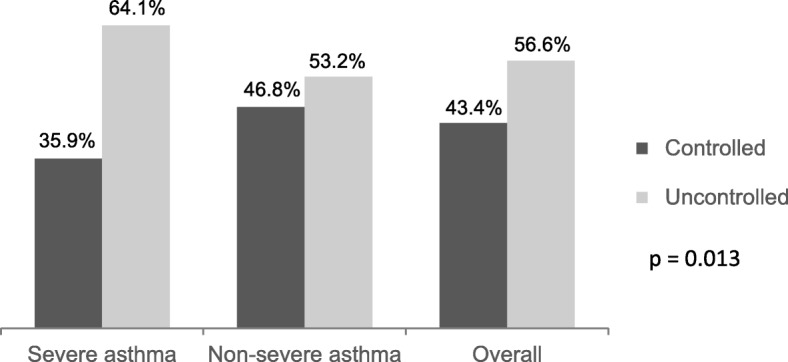
Proportions of controlled and uncontrolled asthma patients in Argentina, Chile, Colombia, and Mexico. 2013–2015

Figure 1 shows that more than a half of SA (64.1%) and NSA (53.2%) patients were uncontrolled, and this proportion was higher in SA when compared to NSA (*p* = 0.013).

Table 3 shows healthcare resources used and the number of clinically significant asthma exacerbation suffered during the last year by SA and NSA patients. Overall, SA patients reported a statistically significant two times higher proportion of both hospital admission (8.7% vs. 3.7%, *p* = 0.011) and almost two times higher proportion for ER visits due to asthma (37.0% vs. 21.7%, *p* < 0.001)) in the last year, compared with NSA patients.

**Table 3:** Asthma exacerbation by severe asthma status in Argentina, Chile, Colombia, and Mexico, 2013–2015

Clinical characteristics	Severe asthma		Non-severe asthma		*p*-value	Total	
	N	(%)	N	(%)		N	(%)
Hospital admissions							
Due to asthma	16	8.7	15	3.7	0.011	31	5.2
Due to other causes	12	6.5	10	2.4	0.015	22	3.7
Hospital admissions mean; median							
Due to asthma	0.17 (0.00)		0.14 (0.00)		0.012	0.15 (0.00)	
Due to other causes	0.12 (0.00)		0.05 (0.00)		0.015	0.07 (0.00)	
Hospital admissions including ICU							
Due to asthma	4	2.2	10	2.4	1.000	14	2.4
Due to other causes	6	3.3	6	1.5	0.150	12	2.0
Hospital admissions including ICU mean; median							
Due to asthma	0.03 (0.00)		0.07 (0.00)		0.839	0.06 (0.00)	
Due to other causes	0.03 (0.00)		0.02 (0.00)		0.152	0.02 (0.00)	
ER visits							
Due to asthma	68	37.0	89	21.7	<0.001	157	26.4
Due to other causes	26	14.1	30	7.3	0.009	56	9.4
ER visits mean; median							
Due to asthma	1.71 (0.00)		0.42 (0.00)		<0.001	0.82 (0.00)	
Due to other causes	0.21 (0.00)		0.12 (0.00)		0.009	0.15 (0.00)	
At least one asthma clinically significant asthma exacerbation	68	37.0	89	21.7	<0.001	157	26.4
Number of clinically significant asthma exacerbation							
0	116	63.0	321	78.3	<0.001	437	73.6
1	22	12.0	45	11.0		67	11.3
2	11	6.0	21	5.1		32	5.4
3	7	3.8	11	2.7		18	3.0
≥ 4	28	15.2	12	2.9		40	6.7
Number of clinically significant asthma exacerbation							
Mean (±SD)	1.88 (±7.85)		0.56 (±2.11)		<0.001	0.97 (±4.74)	
Median (IQR)	0.00 (0)		0.00 (2)			0.00 (1)	

*ER* emergency room, *ICU* intensive care unit

Regarding the hospital admission and emergency visits due to other causes, SA patients also had a significant higher proportion of both events than NSA patients (6.5% vs 2.4%, *p* = 0.015 and 14.1% vs 7.3%, *p* = 0.009). The same pattern was observed when considering the mean number of clinically significant asthma exacerbations (mean 1.88 vs 0.56, *p* < 0.001).

When comparing use of current asthma medication by asthma severity classification (Table 4), the most common medication used in SA patients was ICS (99.5%), even in NSA patients (88.0%). Nevertheless, when comparing combination therapies (Table 5), the most prescribed was ICS/LABA, followed by ICS only in the overall population (49.0 and 17.3%, respectively). The most controller therapy used was ICS/LABA for both SA (47.8%) and NSA (49.5%) patients.

**Table 4:** Self-reported medication used by severe asthma status in Argentina, Chile, Colombia, and Mexico, 2013–2015

	Severe asthma		Non-severe asthma		*p*-value	Overall	
	N	(%)^a^	N	(%)		N	(%)
SAMA	34	18.5	35	8.5	< 0.001	69	11.6
Tiotropium	34	18.5	4	1.0	< 0.001	38	6.4
Antihistamines	21	11.4	22	5.4	0.009	43	7.2
Anti-immunoglobulin E agents	15	8.2	0	–	< 0.001	15	2.5
ICS	183	99.5	361	88.0	< 0.001	544	91.6
High doses	162	88.0	15	3.7		177	29.8
Not high doses	21	11.4	346	84.4		367	61.8
LABA	173	94.0	248	60.5	< 0.001	421	70.9
Leukotriene antagonists	53	28.8	39	9.5	< 0.001	92	15.5
SABA	120	65.2	241	58.8	0.137	361	60.8
Systemic corticosteroids	20	10.9	0	–	< 0.001	20	3.4
Xanthine and adrenergics	10	5.4	6	1.5	0.006	16	2.7
Other asthma medications	0	–	1	0.2	1.000	1	0.2

*SAMA* short-acting muscarinic antagonist, *ICS* inhaled corticosteroids, *LABA* long-acting beta2-agonist, *SABA* short-acting beta2-agonist

^a^The values correspond to the percentage of each drug used and they may be overlapped

**Table 5:** Self-reported combination therapies used by severe asthma status in Argentina, Chile, Colombia, and Mexico, 2013–2015

	Severe asthma		Non-severe asthma		*p*-value	Total	
	N	(%)	n	(%)		N	(%)
ICS + LABA	88	47.8	203	49.5	0.704	291	49.0
ICS only	0	–	103	25.1	< 0.001	103	17.3
ICS + LABA + Tiotropium	8	4.3	0	–	< 0.001	8	1.3
ICS + LABA + LTRA	15	8.2	25	6.1	0.356	40	6.7
ICS + LABA + Xanthine	0	–	2	0.5	1.000	2	0.3
ICS + LABA + Anti-IgE	3	1.6	0	–	0.029	3	0.5
ICS + LTRA	1	0.5	0	–	0.310	1	0.2
ICS + LABA + Antihistamine	9	4.9	10	2.4	0.116	19	3.2
Other	60	32.6	67	16.3	< 0.001	127	21.4

*ICS* inhaled corticosteroids, *LABA* long-acting beta2-agonist, *LTRA* leukotriene receptor antagonists

Finally, when comparing SA with NSA patients regarding self-reported comorbidities (Table 6), the most reported among the SA patients were chronic rhinitis (52.7%) followed by hypertension/hypertension syndrome (32.1%) and gastroesophageal reflux disease (27.2%). On the other hand, among the NSA patients the most frequent reported comorbidities were chronic rhinitis (56.8%), followed by chronic sinusitis and/or rhinosinusitis (22.2%) and gastroesophageal reflux (17.1%). Nevertheless, hypertension, COPD, psychological disturbances (as depression and anxiety), gastroesophageal reflux disease and obesity were higher in SA and this difference was statistically significant.

**Table 6:** Patient-reported comorbidities by severe and non-severe asthma patients in Argentina, Chile, Colombia, and Mexico, 2013–2015

Comorbid Condition	Severe asthma		Non-severe asthma		*p*-value	Total	
	N	(%)	N	(%)		N	(%)
Chronic rhinitis	97	52.7	233	56.8	0.351	330	55.6
Hypertension/hypertension syndrome	59	32.1	55	13.4	< 0.001	114	19.2
Gastroesophageal reflux disease	50	27.2	70	17.1	0.005	120	20.2
Obesity	44	23.9	50	12.2	< 0.001	94	15.8
Chronic sinusitis/rhinosinusitis	31	16.8	91	22.2	0.136	122	20.5
Psychological disturbances (as depression and anxiety disorders)	31	16.8	35	8.5	0.003	66	11.1
Chronic/recurrent respiratory infections	25	13.6	44	10.7	0.315	69	11.6
Hormonal disturbances	18	9.8	28	6.8	0.213	46	7.7
Diabetes	16	8.7	26	6.3	0.301	42	7.1
COPD	11	6.0	10	2.4	0.031	21	3.5
Obstructive sleep apnea/Sleep-disordered breathing	8	4.3	12	2.9	0.375	20	3.4
Smoking	8	4.3	26	6.3	0.333	34	5.7
Cancer^a^	5	2.7	5	1.2	0.298	10	1.7
Cardiac arrythmia^a^	4	2.2	6	1.5	0.509	10	1.7
Ischemic heart disease^a^	4	2.2	6	1.5	0.509	10	1.7
Glottic (vocal cord) dysfunction^a^	2	1.1	6	1.5	1.000	8	1.3
Cerebrovascular disease^a^	1	0.5	1	0.2	0.524	2	0.3
Heart failure^a^	1	0.5	1	0.2	0.524	2	0.3
Other	60	32.6	53	12.9	< 0.001	113	19.0

*COPD* chronic obstructutive pulmonary disease

^a^Fisher Exact Test applied when there were fewer than 5 cases in any cell

## Discussion

To our knowledge, there are no previous description of the SA population in Latin America countries following the updated 2014 ERS/ATS criteria. Results from the present cross-sectional study showed that overall prevalence of SA is 31.0% among patients from outpatient specialized sites in Argentina, Chile, Colombia and Mexico. We found that patients reflecting the ERS/ATS steps 4 and 5 tended to be diagnosed at a later age, had higher proportion of hypertension, COPD, psychological disturbances, gastroesophageal reflux disease, obesity, and experienced increased healthcare utilization.

In our study, we observed that SA patients were older at the time of study and at the time of asthma diagnosis when compared with NSA patients - which is in alignment with other studies [18, 19]. This might be explained by the generalized decline in lung function in older patients, leading to more severe patients [20]. However, our study is limited to reinforce this idea due to lack of a longitudinal analysis and a multivariable analysis controlled by possible confounders.

It is worth noting from our results that despite the use of high doses of ICS in the SA group, more than 60% of them were uncontrolled, proportion that was higher when compared with NSA. Even in the overall population attending those specialized sites, 56.6% were uncontrolled. This was consistent with other studies, where SA patients had an increased number of exacerbations, as well as higher rates of hospital admissions and ER attendances [19].

Current asthma management guidelines acknowledge that PEF monitoring during exacerbations of asthma help determine the severity of these flares and can be useful to guide therapeutic decisions [8]. However, in our study less than 40% in both SA and NSA patients reported using PEF meter at least once in the last year. This low proportion is in accordance with the AIRLA study [10], which evaluated the impact of asthma in Latin America and showed that 54% of asthma patients ever had no spirometry and 96% had not ever performed a PEF test, according to patient self-reported surveys. Although there are differences in methodologies between our study and AIRLA study, which is population-based, both data reinforce that spirometry is not common in Latin America region and the reasons for that should be further evaluated.

As other studies have described that SA patients often display a high number of comorbidities [12, 21], our results found relevant differences between SA and NSA patients with regards to obesity, hypertension, COPD, psychological disturbances and gastroesophageal reflux disease. The SA Research Program (SARP) also showed that hypertension, obesity and gastroesophageal reflux disease are associated with SA [22]. Specially for hypertension, it is a significant predictor for asthma severity, but this relationship was found predominately among the white population, rather than in black patients [23]. It is important to take into account that aging could be a confounding factor in the association found between SA and comorbidities observed in our study. For psychological disturbances, the literature already described higher levels of anxiety and depression in SA patients [24, 25], which is in line with the results found in our study. Finally, COPD has been recently described to be higher in SA group by other authors [26, 27].

The external validity of the current study is limited, as most patients were recruited from specialized asthma clinics and the sample size for ASLA study was not calculated to be representative for each country. Another limitation of our study is the cross-sectional evaluation of medication use, as we did not capture the treatment step up and step down through the past years. The comparison between our result and other studies is difficult, mainly due to the fact that most part of the studies refers to the prevalence of refractory asthma and not to SA. Our analysis may also be subject to recall bias.

## Conclusion

The results of our study may support the identification of SA in our region contributing to the better management of these patients. What is more, this study highlights the need to improve the asthma control in these patients that are already being treated with high ICS doses. Furthermore, complexity of SA phenotypes would require deeper investigations to determine specific phenotypes to be able to prescribe the precise therapy to increase the level of control in SA patients.

## Additional file

Additional file 1: Ethics Committees. All institutional review boards/independent ethics committees. (DOCX 14 kb)

## Data Availability

The datasets used and/or analyzed during the current study are available from the corresponding author on reasonable request.

## Abbreviation

ACT: Asthma control test
Anti-IgE: Anti-immunoglobulin E
ASLA: Asthma control in Latin America study
ATS: American Thoracic Society
BMI: Body mass index
COPD: Chronic obstructive pulmonary disease
ER: Emergency room
ERS: European Respiratory Society
ICS: Inhaled corticosteroids
ICU: Intensive care unit
LABA: Long-acting beta2-agonist
LAMA: Long-acting muscarinic receptor antagonist
LTRA: Leukotriene receptor antagonists
NSA: Non-severe asthma
OCS: Oral corticosteroid
PEF: Peak expiratory flow
SA: Severe asthma
SABA: Short-acting beta2-agonists
USD: United States Dollars
