# Supplementary material for: Asthma severity in four countries of Latin America
Source: BMC Pulm Med. 2019 Jul 9;19:123. doi: 10.1186/s12890-019-0871-1 (PMC6617663; doi:10.1186/s12890-019-0871-1)
Supplement: Supplementary file 1 — Ethics Committees. All institutional review boards/independent ethics committees. (DOCX 14 kb) [file 12890_2019_871_MOESM1_ESM.docx]

**Additional file 1: Ethics Committees names and approval numbers of each site**

| **Investigator** | **Institution** | **Ethics Committee name** | **Ethics Committee approval number** | **Type of institution** |
| --- | --- | --- | --- | --- |
| **Chile** | | | | |
| Dr. Chahuan | Hospital San Borja Arriarán | Comité Ético Científico Servicio de Salud Metropolitano Central | N° 798/13 | Public institution |
| Dr. Galleguillos | Instituto de Medicina Respiratoria | Comité Ético Científico Servicio de Salud Metropolitano Oriente | NA | Public institution |
| Dr. Pavié | Centro Investigaciones Médicas Integrales | Comité Ético Científico Servicio de Salud Viña del Mar-Quillota | NA | Public institution |
| Dr. Bisbal | Hospital Regional Rancagua | Comité Ético Científico Servicio de Salud Metropolitano Oriente | NA | Public institution |
| Dr. Rafael Silva Orellana | Hospital Regional de Talca | Comité Ético Científico Servicio de Salud Metropolitano Oriente | NA | Public institution |
| **Argentina** | | | | |
| Dr Caludio Castaños | Hospital Nacional de Pediatría Garrahan | Comité de Etica en la Investigación Hospital Garrahan | 10-feb-2014 (approval # 802) | Public hospital |
| Dr Daniel Colodenco | Consultorio del Dr Daniel Colodenco | Comité de Etica en Investigacion INAER | 17/fev/14 | Private institution |
| Dr Eduardo Giugno | Centro de Investigación Clínica Belgrano | Comité de Etica en Investigacion INAER | 16/dez/2013 | Private institution |
| Dr Hugo Neffen | Centro de Alergia e Inmunología-Santa Fe- | Comité de Etica en Investigacion INAER | 16/dez/13 | Private institution |
| Dr Gabriel Garcia | CENASMA | Comité de Bioética de la Escuela Latinoamericana de Bioética (CELABE) | 09/jan/14 | Private institution |
| **Colombia** | | | | |
| Dr. Álvaro Burbano | IPS Caja de Compensación Familiar | Comité Institucional de Ética en Investigación C.I.E.I CAFAM | CIEI/2013-1311 | Private Institutions/Hospitals |
| Dr. Fabio Bolivar | Instituto Neumológico del Oriente | Comité de Ética en Investigación Biomédica Instituto Neumológico del Oriente (CEINO) | NA | Private Institutions/Hospitals |
| Dr. Rubén Contreras | Clínica Colombia COLSANITAS | Comité de Ética en Investigación Fundación Universitaria Sanitas | CEIFUS 1755-14 | Private Institutions/Hospitals |
| **Mexico** | | | | |
| Dr. Sanches | Unidad de Investigación en Salud de Chihuahua, S.C. | Unidad de Investigación en Salud en Chihuahua, S.C. | UIS-14-010 | Private Intitution |
| Dr. Edith Vallejo Perez | Unidad de Investigación Respiratoria de Michoacán | Instituto Jalisciense de Investigación Clínica, S.A. de C.V. | 00458 | Private Intitution |
| Dr. Dante Colin | Instituto Jalisciense de Investigación Clínica | Instituto Jalisciense de Investigación Clínica, S.A. de C.V. | 00432 | Private Intitution |
